# Investigating mental workload-induced changes in cortical oxygenation and frontal theta activity during simulated flights

**DOI:** 10.1038/s41598-022-10044-y

**Published:** 2022-04-19

**Authors:** Anneke Hamann, Nils Carstengerdes

**Affiliations:** Deutsches Zentrum für Luft- und Raumfahrt e.V. (DLR), Institut für Flugführung, Lilienthalplatz 7, 38108 Braunschweig, Germany

**Keywords:** Cognitive neuroscience, Psychology

## Abstract

Monitoring pilots’ cognitive states becomes increasingly important in aviation. Physiological measurement can detect increased mental workload (MWL) even before performance declines. Yet, changes in MWL are rarely varied systematically and few studies control for confounding effects of other cognitive states. The present study targets these shortcomings by analysing the effects of stepwise increased MWL on cortical activation, while controlling for mental fatigue (MF). 35 participants conducted a simulated flight with an incorporated adapted n-back and monitoring task. We recorded cortical activation with concurrent EEG and fNIRS measurement, performance, self-reported MWL and MF. Our results show the successful manipulation of MWL without confounding effects of MF. Higher task difficulty elicited higher subjective MWL ratings, performance decline, higher frontal theta activity and reduced frontal deoxyhaemoglobin (Hbr) concentration. Using both EEG and fNIRS, we could discriminate all induced MWL levels. fNIRS was more sensitive to tasks with low difficulty, and EEG to tasks with high difficulty. Our findings further suggest a plateau effect for high MWL that could present an upper boundary to individual cognitive capacity. Our results highlight the benefits of physiological measurement in aviation, both for assessment of cognitive states and as a data source for adaptive assistance systems.

## Introduction

The assessment of cognitive states and resources has long been of interest in aviation research and dates back to the 1970s^1^. While the current focus of aviation research points towards higher levels of automation, humans are still considered a vital part in the future of aviation^2^. New technologies such as intelligent and adaptive assistance systems are hoped to improve safety and efficiency in day-to-day operations by tailoring assistance to the human operator’s needs. Yet, these systems need information on the operator’s current cognitive state. Physiological measurement is considered an essential data source for real-time assessment^3^. This type of measurement has some advantages over simple performance measurement and self-report: With its higher temporal resolution it is possible to capture changes in cognitive states even before performance is affected. In addition, it is more objective than self-report, not biased by social desirability or the person’s ability for introspection.

The cognitive state that is of particular interest in aviation research and physiological real-time assessment is mental workload (MWL)^3,4^. It is defined as the part of one’s cognitive resources that is required to perform the current task^5^. The more demanding the task, the more resources need to be allocated to performing it. Cognitive resources, however, are limited and exceeding one’s limits may lead to reduced performance and, in turn, errors^6,7^. MWL can be assessed using, among others, electroencephalography (EEG) and functional near-infrared spectroscopy (fNIRS). EEG band power analyses allow for a continuous monitoring of MWL. Especially theta and alpha band activity have been studied extensively, as well as a combination of both bands, in ratios^8–10^ or head maps^11^, in order to increase sensitivity to changes. Theta activity at frontal electrode sites typically increases with rising MWL^12–14^, while alpha and beta activity at parietal sites are usually reduced^15,16^. EEG-based MWL assessment has been used in driving^17^, flying^14,16^, and air traffic control^11^ tasks. In recent years, fNIRS has been proposed as an alternative or addition to EEG because of its comparatively lower susceptibility to motion artefacts and higher spatial resolution^18^. fNIRS measurement relies on a change in oxygen concentration in cerebral blood flow that indicates higher brain activity in the measured region. This way, differences in cognitive demands can be distinguished: Higher MWL leads to higher (pre-) frontal cortex activation, as indicated by higher concentrations of oxyhaemoglobin (Hbo), lower concentrations of deoxyhaemoglobin (Hbr), and higher total haemoglobin (Hbt)^18^. These effects have been observed both for basic working memory tasks^19–23^ as well as applied tasks including driving^24–26^, UAV and air traffic control^27^, and flying an aircraft^28,29^.

Before developing assistance systems relying on physiological MWL assessment, however, one should consider the influence of other cognitive states on brain activity. One important state is mental fatigue (MF). It is described as the state between high alertness and sleepiness, and characterized by the desire to avoid further effort^30^. It is typically induced by continuous task execution and can eventually lead to sleepiness if not counteracted, e.g. by taking breaks^30,31^. In EEG, growing MF is associated with increasing frontal theta activity^32,33^, similar to the effects of rising MWL. While this poses a problem for valid assessment and discrimination of MWL and MF in experimental settings, the implications for the application in the cockpit remain the same: Frontal theta activity increases with rising cognitive demand, regardless of its nature. Parietal alpha and beta activity, however, rise with growing MF^33–35^, contrary to effects of MWL. This opposing trend in alpha and beta power, depending on which cognitive state is present, poses a problem for the validity of the assessment of either state if the other one is not controlled for. An interaction of MWL and MF may weaken or completely suppress any observable alpha or beta effect^15,32^. Therefore, MWL indices relying on alpha, beta or ratios including these bands may lose meaningfulness over longer periods of continuous work when MF accumulates. In fNIRS, MF is characterized by an increase in frontal Hbo and decrease in frontal Hbr over time^35^, as early as 20 min after task onset^36^. Such elevated cortical activation is thought to counteract the effects of MF^37^. Thus, cortical oxygenation increases both with MWL and MF, similar to the observations on frontal theta activity in EEG. To date, there is only a handful of studies that have focused on the interaction of cognitive states and its effects on measurement validity in EEG^32,38–40^. To our knowledge, no such studies have been published for fNIRS. Furthermore, few studies explicitly control for confounding cognitive states when manipulating MWL.

In the present study we targeted this problem by assessing physiological correlates of MWL in EEG and fNIRS while controlling for the influence of MF. We used an Airbus A321 flight simulator and tailored the experimental tasks to the aviation context. In order to induce different levels of MWL, we employed an adapted n-back task^41^ in four difficulty levels (0–3-back). In the classical n-back task, participants are given a sequence of stimuli and have to decide if the current stimulus is the same as the one n steps back^42^. This way, the n-back places demands on working memory capacity because it requires a continuous monitoring and updating of information^43^. The possibility to increase task difficulty stepwise by increasing n makes the task an ideal choice for manipulation of MWL. The adapted n-back task incorporates elements of a digit-span task^41^: The participants have to memorize and reproduce targets in the correct order instead of a simple recall. This way, the chance of guessing is minimized. We adapted the task further to fit the aviation context by using auditory heading commands that served as stimuli, and a parallel monitoring task in which the participants had to monitor and correct the altitude of the aircraft. This resulted in a 4 (n-back level) × 2 (first, second presentation of each n-back level) study design. We took measures to control for the influence of MF by keeping the task to 40 min (as MF is found to manifest after approx. 45–70 min of task execution^33,44^), pseudo-randomizing the different n-back levels to prevent time-on-task effects from coinciding with MWL and by testing for differences between the two presentations of each n-back level. Additionally, we used self-report (KSS^45^, F-ISA^46^) to assess MF. In order to control for experience and physiological variations due to age^47^, we chose a student sample without prior flying experience. We assessed MWL with EEG, fNIRS, performance measures and self-report (ISA^48^). We hypothesised that no MF would be induced during the experiment, and that higher MWL would manifest in higher frontal theta and lower parietal alpha and beta activity in EEG, higher oxygenation changes in frontal regions in fNIRS, reduced task performance, and higher subjective MWL ratings.

## Results

### Time-on-task effects

Analyses showed no time-on-task effects for the majority of measures, indicating time on task did not coincide with mental workload. Bonferroni-corrected paired t-tests did not show any significant difference between the first and second presentation of each n-back level, *p* > 0.05, except for the subjective mental fatigue assessment (F-ISA), all *p*s ≤ 0.012. The variable “presentation” was therefore removed from further analyses, except for F-ISA.

### Subjective data

**Figure 1:**
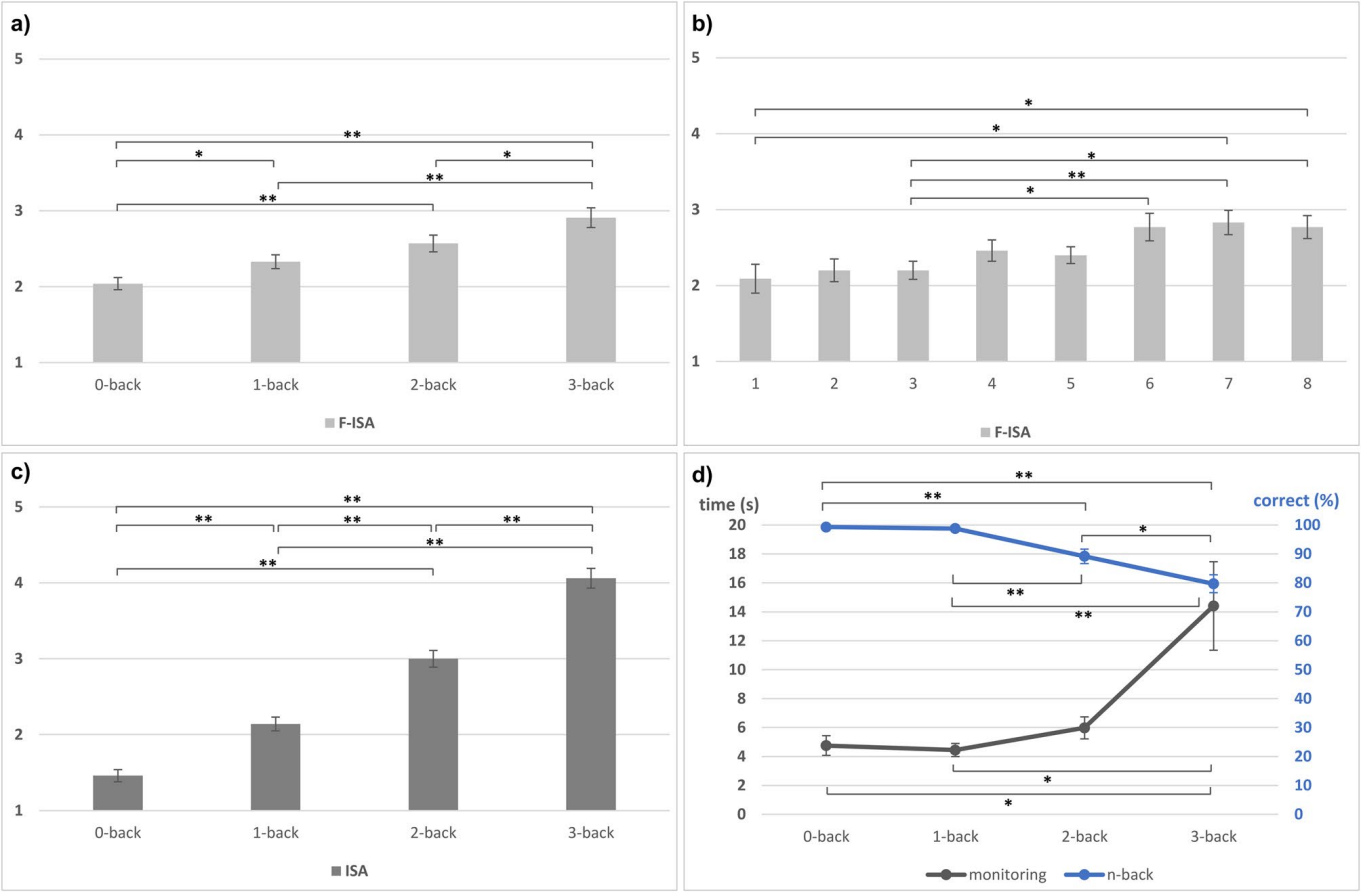
Subjective and performance measures. Error bars indicate SE. Significant comparisons marked *p* < 0.05* and *p* < 0.01**. (**a**) Mean F-ISA ratings per n-back level. (**b**) Mean F-ISA ratings across experimental blocks. (**c**) Mean ISA ratings per n-back level. (**d**) Mean monitoring and n-back performance per n-back level.

Using subjective data, all four n-back levels could be discriminated, see Table 1. Analyses on the influence of task difficulty showed substantial effects, both on mental workload (ISA) and mental fatigue (F-ISA), see Fig. 1a,c respectively. The ANOVA on mental workload revealed a significant effect of n-back level on ISA, *F*(3, 102) = 212.89, *p* < 0.001, η^2^_p_ = 0.86. Concerning mental fatigue, the ANOVA showed a significant effect of n-back level on F-ISA, *F*(3, 102) = 19.30, *p* < 0.001, η^2^_p_ = 0.36 and a significant effect of presentation, *F*(1, 34) = 28.69, *p* < 0.001, η^2^_p_ = 0.46. The interaction was not significant, *p* > 0.05. Post-hoc comparisons showed significant differences between all n-back levels except 1- versus 2-back, all *p*s < 0.001, and between the two presentations of each n-back level, *p* < 0.001, see Table 2.

**Table 1:** Overview of different MWL measures and their ability to discriminate the four n-back levels.

n-back level comparison	Self-report	Performance		EEG—theta activity			fNIRS—Hbr, ROIs	
	ISA	Monitoring	n-back	Fz	F3	F4	Left	Right
**0 versus**								
1	*✓*						*✓*	*✓*
2	*✓*		*✓*	*✓*	*✓*	*✓*	*✓*	*✓*
3	*✓*	*✓*	*✓*	*✓*	*✓*	*✓*	*✓*	*✓*
**1 versus**								
2	*✓*		*✓*	*✓*	*✓*		*✓*	*✓*
3	*✓*	*✓*	*✓*	*✓*	*✓*	*✓*	*✓*	*✓*
**2 versus**								
3	*✓*		*✓*	*✓*				

Significant differences (*p* < 0.05) marked with ✓. EEG alpha and beta activity are omitted as no significant comparisons were found.

**Table 2:** Bonferroni-corrected post-hoc pairwise comparisons for subjective and performance measures.

Measure	Factor	Comparison		Mean difference (SE)	*p*-value
F-ISA	n-back level	0 versus	1	− 0.29 (0.09)	0.019*
			2	0.53 (0.11)	< 0.001**
			3	− 0.87 (0.14)	< 0.001**
		1 versus	2	− 0.24 (0.11)	0.180
			3	− 0.59 (0.14)	0.001**
		2 versus	3	− 0.34 (0.12)	0.041*
	Presentation	1 versus	2	− 0.41 (0.08)	< 0.001**
ISA	n-back level	0 versus	1	− 0.69 (0.09)	< 0.001**
			2	− 1.54 (0.11)	< 0.001**
			3	− 2.60 (0.12)	< 0.001**
		1 versus	2	− 0.86 (0.09)	< 0.001**
			3	− 1.92 (0.12)	< 0.001**
		2 versus	3	− 1.06 (0.12)	< 0.001**
Monitoring (time in s)	n-back level	0 versus	1	0.31 (0.55)	> 0.999
			2	− 1.22 (0.95)	> 0.999
			3	− 9.66 (3.20)	0.030*
		1 versus	2	− 1.54 (0.69)	0.204
			3	− 9.97 (3.14)	0.020*
		2 versus	3	− 8.44 (3.22)	0.081
n-back (% correct)	n-back level	0 versus	1	0.5 (0.6)	> 0.999
			2	10.1 (2.4)	0.001**
			3	19.6 (3.1)	< 0.001**
		1 versus	2	9.5 (2.2)	0.001**
			3	19.1 (3.3)	< 0.001**
		2 versus	3	9.5 (3.2)	0.028*

Significance levels marked *p* < 0.05* and *p* < 0.01**.

Analyses on the influence of time on task on mental fatigue showed small, but substantial increases over time. Mean subjective sleepiness (KSS) before the experiment was 2.63 (“alert”; *SD* = 0.97), and 4.74 (“neither alert nor sleepy”; *SD* = 1.67) after the experiment. A paired t-test showed a significant difference, *t*(34) =−7.71, *p* < 0.001. Mean mental fatigue (F-ISA) increased by 0.69 points between block 1 and 8, from 2.09 (“low”) to 2.77 (“medium/relaxed wakeful”), see Fig. 1b. The ANOVA showed a significant effect of time on task on F-ISA, *F*(4.90, 166.47) = 6.65, *p* < 0.001, η^2^_p_ = 0.16. Bonferroni-corrected post-hoc pairwise comparisons showed significant differences between block 1 and 7 as well as 8 (all *p*s ≤ 0.023), and between block 3 and blocks 6, 7 and 8 (all *p*s ≤ 0.015).

### Performance data

The two performance measures showed different potential to discriminate the four n-back levels, see Table 1. Analyses on the influence of task difficulty on performance showed substantial performance declines with increasing difficulty, see Fig. 1d. For monitoring, the ANOVA revealed a significant effect of n-back level on reaction time, *F*(1.15, 35.66) = 8.33, *p* = 0.005, η^2^_p_ = 0.21. Post-hoc comparisons showed significant differences between 3-back and 0- and 1-back, respectively, all *p*s ≤ 0.030, see Table 2. For n-back performance, the ANOVA revealed a significant effect of n-back level on correct responses, *F*(1.86, 63.28) = 24.95, *p* < 0.001, η^2^_p_ = 0.42. Post-hoc comparisons showed significant differences between all n-back levels but 0-back versus 1-back, all *p*s ≤ 0.028, see Table 2.

### EEG data

Using EEG Power Spectral Density, all n-back levels could be discriminated apart from the two lowest levels, albeit only in frontal theta activity, see Table 1. For theta activity, the MANOVA indicated a significant multivariate effect on the three frontal electrodes (Fz, F3, F4), Pillai’s trace *V* = 0.70, *F*(9, 26) = 6.58, *p* < 0.001, η^2^_p_ = 0.70. Univariate ANOVAs revealed significant differences for all three frontal sites, electrode Fz, *F*(1.56, 52.87) = 23.91, *p* < 0.001, η^2^_p_ = 0.41, electrode F3, *F*(1.71, 52.87) = 12.56, *p* < 0.001, η^2^_p_ = 0.27, and electrode F4, *F*(3, 102) = 14.64, *p* < 0.001, η^2^_p_ = 0.30. See Table 3 for post-hoc comparisons and Supplement Fig. S1 for visualisation.

**Table 3:** Bonferroni-corrected post-hoc pairwise comparisons for theta activity (Power Spectral Density in µV^2^/Hz) at frontal electrodes.

Electrode	Comparison		Mean difference (SE)	*p*-value
Fz	0 versus	1	− 0.02 (0.01)	0.055
		2	− 0.08 (0.01)	< 0.001**
		3	− 0.15 (0.03)	< 0.001**
	1 versus	2	− 0.05 (0.01)	< 0.001**
		3	− 0.12 (0.02)	< 0.001**
	2 versus	3	− 0.07 (0.02)	0.023*
F3	0 versus	1	− 0.01 (0.01)	> 0.999
		2	− 0.06 (0.01)	< 0.001**
		3	− 0.10 (0.02)	0.002**
	1 versus	2	− 0.05 (0.01)	0.001**
		3	− 0.09 (0.02)	0.002**
	2 versus	3	− 0.04 (0.02)	0.421
F4	0 versus	1	− 0.02 (0.01)	0.204
		2	− 0.06 (0.02)	0.005*
		3	− 0.09 (0.02)	< 0.001**
	1 versus	2	− 0.03 (0.01)	0.142
		3	− 0.07 (0.02)	0.001*
	2 versus	3	− 0.03 (0.02)	0.200

Significance levels marked *p* < 0.05* and *p* < 0.01**.

The MANOVA for parietal alpha activity did not yield any significant results.

For beta activity, the MANOVA indicated a significant multivariate effect on the three parietal electrodes (Pz, P3, P4), Pillai’s trace *V* = 0.59, *F*(9, 26) = 4.16, *p* = 0.002, η^2^_p_ = 0.59. Univariate ANOVA revealed significant differences only for electrode P3, *F*(2.13, 72.41) = 4.48, *p* = 0.013, η^2^_p_ = 0.12. Bonferroni-corrected post-hoc pairwise comparisons did not show any significant differences between n-back levels.

### fNIRS data

Using Hbr, all n-back levels could be discriminated apart from the two highest levels, see Table 1. We focus on results in Hbr as it is found to be less influenced by systemic noise^49,50^ and to correlate higher with the BOLD signal^51^ than Hbo. In addition, there is evidence that by using a GLM approach and analyzing beta coefficients, Hbr outperforms Hbo in discriminating multiple n-back difficulties and MWL levels in a simulated driving task^20,24^. Hbo results are included in Supplement Table S1.

**Figure 2:**
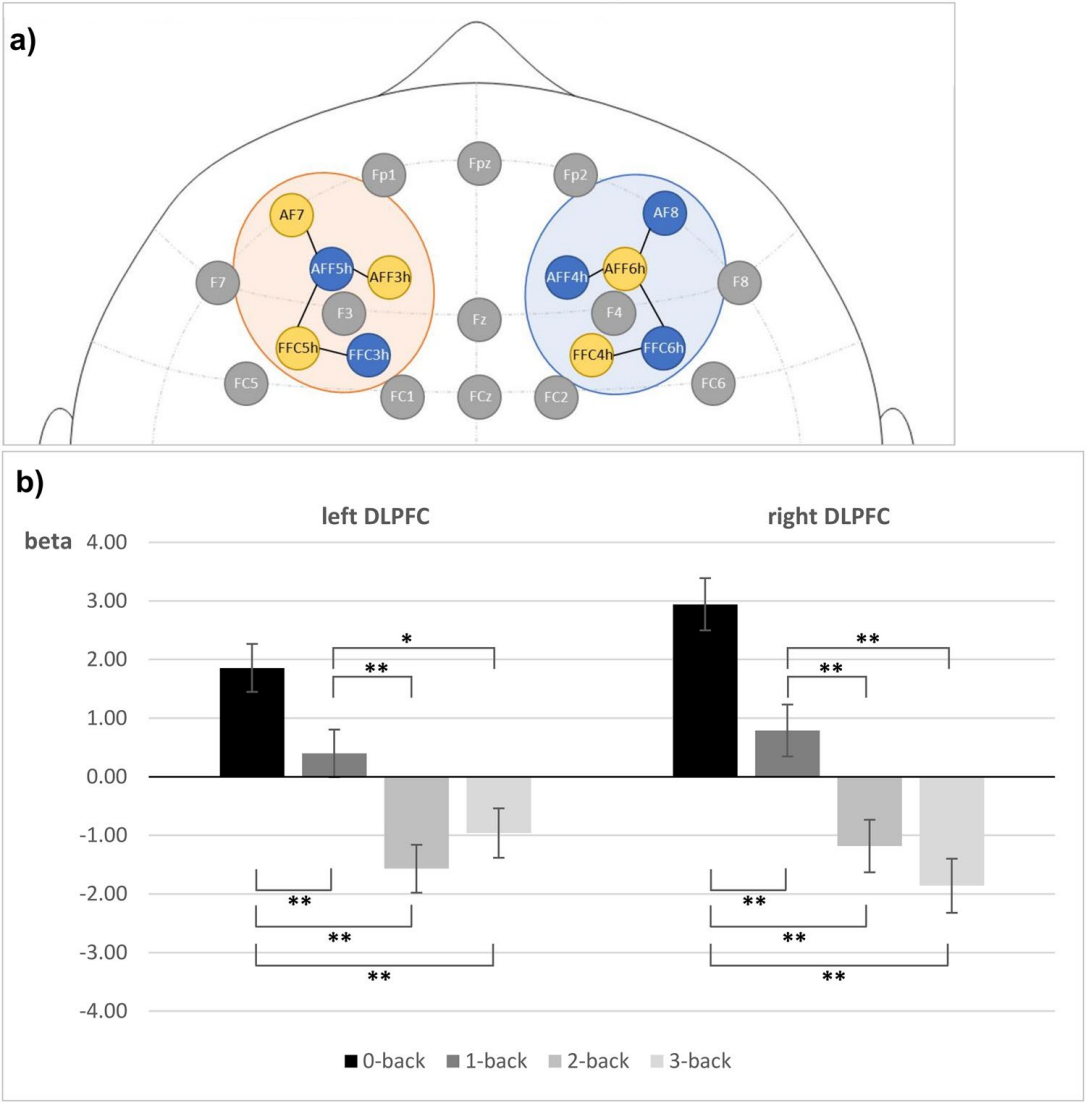
(**a**) fNIRS channels used to build the ROIs. (**b**) Beta values for Hbr per ROI and n-back level. Error bars indicate SE. Significance levels marked *p* < 0.05* and *p* < 0.01**.

Channel-wise t-contrasts showed 24 significant contrasts in nine different channels for Hbr: four on the left and four on the right hemisphere in mirrored locations, as well as an additional fifth channel on the right, see Supplement Table S2 and Supplement Fig. S1. Based on these results, we built two ROIs, left and right dorsolateral prefrontal cortex (DLPFC), consisting of the mirrored four significant channels per hemisphere, see Fig. 2a. Both ROIs showed significant contrasts, see Table 4 and Fig. 2b.

**Table 4:** Haemodynamic results for ROI t-contrasts in Hbr.

Comparison	ROI	β (SE)	*t*	*p*-value
**0 versus**				
1	Left	− 1.46 (0.50)	− 2.94	0.009**
	Right	− 2.15 (0.55)	− 3.93	< 0.001**
2	Left	− 3.43 (0.51)	− 6.84	< 0.001**
	Right	− 4.13 (0.55)	− 7.44	< 0.001**
3	Left	− 2.82 (0.51)	− 5.53	< 0.001**
	Right	− 4.80 (0.57)	− 8.49	< 0.001**
**1 versus**				
2	Left	− 1.97 (0.50)	− 3.97	< 0.001**
	Right	− 1.97 (0.55)	− 3.56	< 0.001**
3	Left	− 1.36 (0.51)	− 2.69	0.012*
	Right	− 2.65 (0.56)	− 4.70	< 0.001**
**2 versus**				
3	Left	0.61 (0.51)	1.19	0.257
	Right	− 0.68 (0.57)	− 1.19	0.315

β = coefficient of the regression model.

FDR-corrected *p*-values^52^. Significance levels marked *p* < 0.05* and *p* < 0.01**.

## Discussion

In the present study we aimed at finding valid physiological markers of MWL in a simulated flight task. Therefore, we applied two parallel tasks tailored to the aviation context, an adapted n-back task in four difficulty levels and a parallel monitoring task, while controlling for the influence of MF by means of task duration and randomization.

Concerning the manipulation of MWL, subjective MWL ratings (ISA) increased substantially with increasing n-back level, pointing towards a successful induction of four distinct difficulty levels.

Concerning the control of MF by means of task duration, our analyses suggest a low influence on MWL. We did not find any substantial difference between the first and second presentation of each n-back level in any measure, apart from the subjective MF rating (F-ISA), indicating no general time-on-task effect and thereby a prevention of coinciding MWL and MF. F-ISA ratings increased both with increasing n-back level and between the two presentation of each n-back level, but no significant interaction could be found. Further investigation of the time-on-task effect on F-ISA also showed a general increase across blocks. KSS ratings increased similarly between the beginning and end of the experiment. However, while significant, the increase in MF can be considered minor, as both measures indicate a shift from an alert to a neutral, yet not fatigued state. In sum, while the accumulation of MF across time cannot be ruled out completely, both subjective MF measures showed only minor increases over time and no time-on-task effects could be found for any other measure. We thus conclude that the task duration of 40 min in combination with the randomization of n-back levels prevented MF from confounding with MWL.

The n-back performance showed a ceiling effect for both 0- and 1-back, but decreased linearly with higher n-back levels, by about 10% with each n-back step. In the 3-back condition, mean accuracy was still at about 80%, indicating that most participants could maintain their performance for the duration of the task. Monitoring performance dropped significantly in the 3-back condition. The time until a reaction was initiated approx. tripled in comparison with the other n-back levels. We interpret this as an indication that cognitive capacity limits were reached and that the participants had to re-allocate resources^53,54^. In order to keep up the n-back performance, they seemed to de-prioritize the monitoring task. We therefore conclude that the 3-back in combination with the monitoring task was challenging enough to lead to cognitive overload. Interestingly, the self-reported MWL level suggested high, but not excessive load in this condition.

Using EEG, we could discriminate all n-back levels apart from 0- versus 1-back. In accordance with previous research^13,39,55^, frontal theta activity was sensitive to MWL changes. The electrode Fz outperformed the electrodes F3 and F4 in the ability to differentiate the n-back levels. Given that a smaller set of sensors will benefit the integration of physiological measurement into the cockpit, we refrained from additionally combining the electrodes into a frontal ROI. Contrary to other findings^15,16,38^, alpha activity did not show any significant change. This could be due to confounding factors: There is evidence that alpha and beta activity increase with time on task and decrease with MWL^32^. If both states are present, alpha and beta effects might be cancelled out, thereby reducing classification accuracy^32^. However, the randomization of n-back levels across time should have accounted for such time-on-task effects in the present study, and no differences could be detected between the two presentations of each n-back level. Therefore, a second explanation for the lack of changes in alpha power seems more plausible: Task switching. Researchers found higher alpha activity with frequent task switching^13^. They hypothesized that alpha activity reflects strategy use and therefore confounds with task demands, while theta activity reflects MWL. In our experiment, the participants had to change headings and monitor and adjust the flight level of the simulated aircraft in parallel, which might have induced similar task switching effects and prohibited alpha suppression with higher MWL levels. This should be investigated further as real flight situations also require the pilots to complete multiple tasks in parallel. Beta activity showed significant changes, but post-hoc comparisons could not reveal any significant differences between difficulty levels. This aligns with previous, ambiguous results on the beta band. While it sometimes is considered the best indicator for MWL^12^, others discussed if beta reflected types of cognitive processing instead of load^55^. As stated above, the beta band is also influenced by time-on-task effects^32^. Taken together, for MWL assessment in a task like ours we do not consider the alpha and beta bands reliable markers as they seem susceptible to interference from other states and task characteristics. We suggest focusing on frontal theta activity for MWL discrimination in applied settings as it seems the most robust EEG measure. Especially the measurement at the electrode position Fz seems most promising for future use in real-life aircraft operations.

Looking at cortical oxygenation changes in Hbr using fNIRS, we found a mirrored pattern of four channels per hemisphere that showed significant differences between difficulty levels. Higher task difficulty was generally associated with lower Hbr concentrations, adding to previous results on Hbr in n-back tasks^20,21^. Discrimination was better in the right DLPFC as compared to the left, in line with previous findings^20^. However, channel sensitivity changed with the comparison of different difficulty levels and no single channel could discriminate all four levels. These variations in channel sensitivity might be due to higher variability because of the influence of artefacts on single channels. When combined into two ROIs to obtain more robust results, differentiation was possible between all n-back levels apart from the two highest levels (2- vs. 3-back). We therefore suggest that using fNIRS at least three different MWL levels can be differentiated.

When comparing behavioural and physiological results, the ceiling effect in performance between 0- and 1-back aligns with the inability to differentiate both levels using EEG, indicating that the demands on cognitive resources were not strong enough to either elicit significant changes in frontal theta activity or impact performance. In comparison, changes in frontal Hbr concentration seem more sensitive to low MWL as differentiation was possible in both ROIs. Interestingly, the sudden performance decrease in monitoring between 2- and 3-back was not as distinct in physiology, as neither EEG nor fNIRS could consistently differentiate both levels: In fNIRS, we did not observe any significant difference, while in EEG only theta activity at electrode Fz differed significantly between the two levels. One could argue that neither EEG nor fNIRS are sensitive enough to small MWL changes and can only consistently differentiate low (0-, 1-back) from high (2-, 3-back) MWL. In fact, differentiation between non-adjacent n-back levels has been reported as better than between adjacent levels^20,21^. However, our results suggest that more than two difficulty levels can be differentiated, and such observations have previously been made both for EEG^11,13^ and fNIRS^25,41^. Alternatively, it is possible that physiological changes reached a plateau after the 2-back condition and a further increase of the difficulty level could not be compensated, thus leading to the observed performance decline. Previous research found such a plateau effect in (pre-) frontal oxygenation changes in fNIRS^19,56^ and EEG theta activity^13^ for tasks with high difficulty. The authors interpreted the plateau as an individual processing capacity limit that also limited performance, depending on the participants’ individual cognitive resources. If such a limit is indeed there and can be assessed using EEG and fNIRS, this would be of great interest for detection of overload and an upper boundary for individual cognitive capacity.

Taken together, we found clear evidence that different MWL levels induced by the n-back task could be distinguished using performance, self-report and both EEG and fNIRS. Furthermore, a combination of EEG and fNIRS may prove useful as frontal Hbr concentration was more sensitive to tasks with low difficulty, while frontal theta activity may better discriminate tasks with higher difficulty. Physiological measures clearly show the potential to assess MWL during simulated flight tasks, and possibly to define individual cognitive capacity limits. This is also an interesting property for future adaptive assistance systems. In order to tailor assistance to the human operator’s needs, such systems need to be able to differentiate cognitive states, account for interactions and, if possible, detect individual capacity limits. While there is still a long way to having fully functional adaptive assistance systems in aviation, valid physiological measures are key to achieving this goal.

## Methods

### Sample

Of 38 volunteers, three were excluded due to insufficient performance after practice. Therefore, the final sample comprised 35 participants (24 male, 11 female) aged 19–30 years (*M* = 23.7, *SD* = 2.1). All fulfilled the following inclusion criteria: They were students, German native speakers, right-handed, had normal or corrected-to-normal vision and no previous flying or flight simulator experience. The participants had been instructed to follow their usual sleep and caffeine habits prior to the experiment. They provided written informed consent and received monetary compensation of 25 €. The study was approved by the ethics commission of the German Psychological Society (DGPs) and conducted in accordance with the declaration of Helsinki.

### Flight simulation

**Figure 3:**
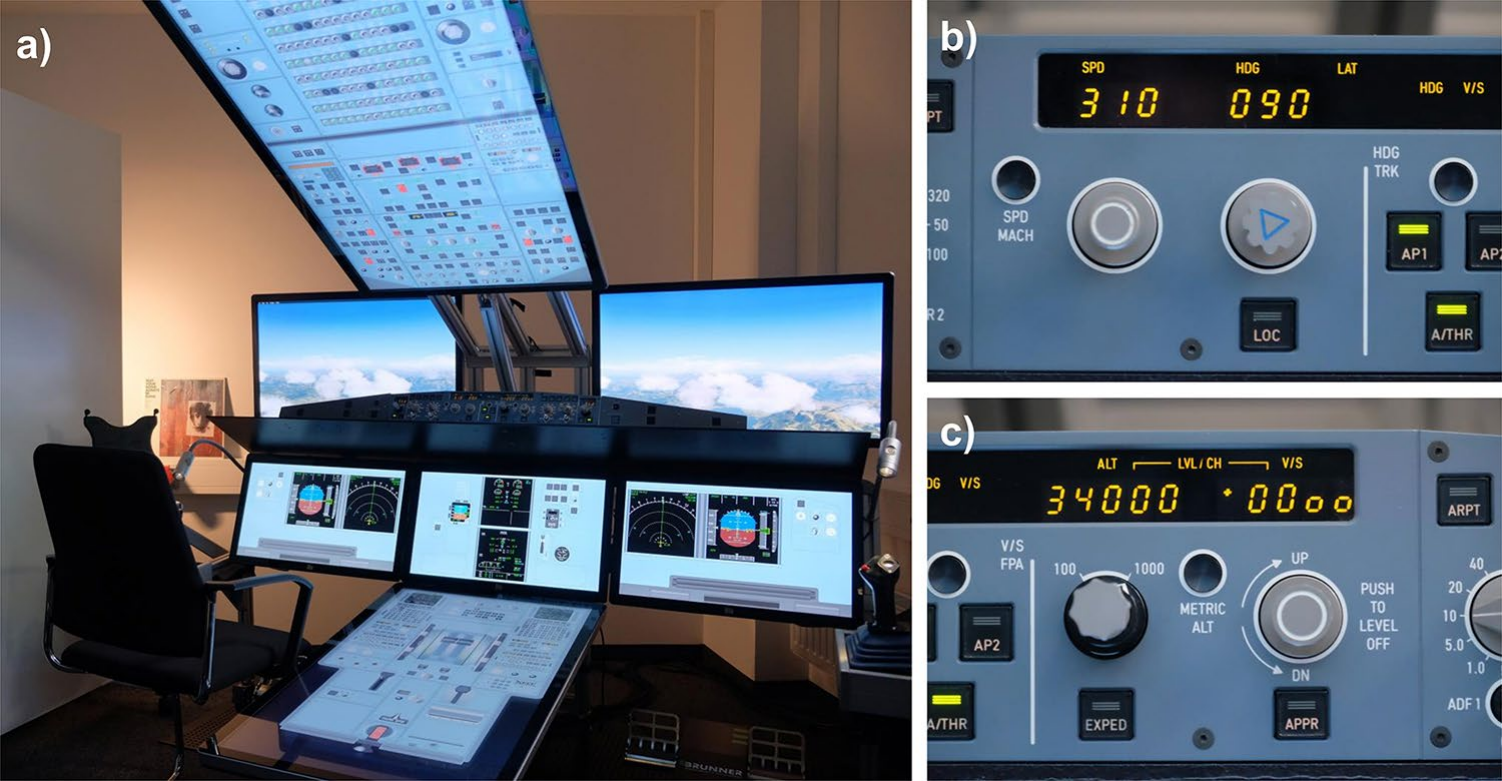
(**a**) The iSim runs on X-Plane 11 and consists of two displays for the outside view, six touch displays for the cockpit instruments, and hardware components for glareshield, side sticks and pedals. (**b**) Close-up of the heading dial. (**c**) Close-up of the vertical speed dial.

We used the flight simulator iSim of the Institute of Flight Guidance at DLR Braun-schweig. The iSim was configured as an Airbus A321 cockpit for this study (see Fig. 3). The flight task was designed for one pilot (i.e. single-pilot cockpit). Only cruise flight was simulated and most functions were controlled by the autopilot in order to create an easy flight task that did not require any flight experience. Participants were seated on the left side of the cockpit. They monitored the primary flight display and should only operate the heading dial and vertical speed dial, see Fig. 3b,c).

### Experimental task and material

The experimental task consisted of eight blocks lasting approx. 3 min each, in which the participants completed two parallel tasks. After each block participants were asked to rate their MWL and MF, followed by a 2-min rest period. In total, the experimental task lasted approx. 40 min.

#### Monitoring task

The aircraft altitude was set at 20,000 ft. Participants had to monitor the altitude and correct deviations greater than 40 ft as fast as possible. No time limit was given. The experimenter triggered one deviation per block. The onset was fixed within each block, but randomized across blocks.

#### Adapted n-back task

We created an auditory version of the adapted n-back task developed by Unni et al.^41^ that fit the aviation context (in the following referred to as “n-back task”) to manipulate working memory load in four levels (0–3-back). The participants had to change the heading of the aircraft according to auditory heading commands. The heading of an aircraft describes its current course in degree on a 360° compass, e.g. a heading of 090 equals an eastward course and a heading of 270 a westward course. The participants followed the heading commands in line with the current n-back level, i.e. set the heading announced n prior to the current command. The task was programmed in PsychoPy3 v2020.1^57^. At the beginning of each block the difficulty level of the n-back task was announced. Each difficulty level was presented twice in a pseudo-randomized order so that the same level was never presented in consecutive blocks, resulting in at least ten minutes between the two presentations. In every block the initial heading was set to 270 and a sequence of eight discrete heading commands was given, each followed by a random inter-stimulus interval (*M* = 22 s, *SD* = 3 s) to avoid confounding with Mayer waves in the fNIRS signal^58,59^. For each participant, the heading sequences and n-back levels were matched randomly.

#### Subjective measures

The KSS^45^ in the German translation^60^ was used to assess general level of sleepiness before and after the experiment on a scale from 1 (“extremely alert”) to 9 (“very sleepy”). After each block, subjective MWL and MF ratings were given verbally. MWL was assessed with the ISA^48^ on a 1–5 scale (“underutilized” to “excessive workload”). MF was assessed with the F-ISA^46^ on a 1–5 scale (“very low/alert” to “very high/fatigued”).

### Procedure

The participants completed a demographic questionnaire, the first KSS assessment, and received instructions about the iSim and the tasks. They then practiced both tasks in parallel. The difficulty levels of the n-back task were presented in ascending order (0–3-back). To avoid learning effects the initial heading during practice was set to 090. Participants needed to achieve min. 60% correct reactions to move to the next difficulty level, and had up to three tries per level. Feedback on both tasks was given during the practice session, but not during the main task. After practising, concurrent EEG and fNIRS recordings were prepared and calibrated and the participants moved on to the main task. After completion of the main task, participants gave the second KSS rating, were thanked and compensated.

### Physiological data recording

**Figure 4:**
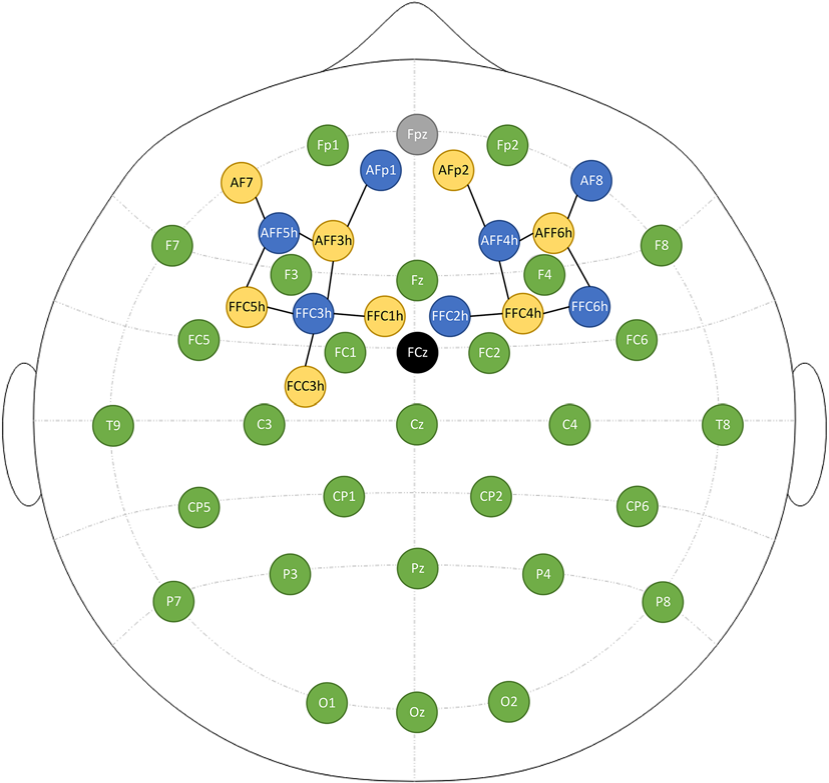
Combined fNIRS-EEG montage. Colours: Blue = fNIRS detector; yellow = fNIRS source incl. short distance channel; green = electrode, grey = ground electrode, black = reference electrode.

EEG signals were recorded at 500 Hz with a LiveAmp-32 device and in BrainVision Recorder 1.23 (Brain Products GmbH, Gilching, Germany). fNIRS signals were recorded at 10 Hz with an eight-source/seven-detector (plus eight additional short-distance channels) time-multiplexed dual-wavelength NIRSport2 device using Aurora 2020.7 (NIRx Medical Technologies LLC, Glen Head, NY, USA). 28 Ag/AgCl active EEG electrodes were positioned according to the 10–20 system with online reference at FCz and fNIRS optodes were positioned in between in a custom montage (see Fig. 4). Optode positioning was determined with the fNIRS Location Decider fOLD v2.2^61^, using the AAL2 brain atlas for coverage of the middle frontal gyrus. Seven channels with a specificity of 47.4% or higher were obtained. As the fOLD solution did not exhaust the number of optodes available, the remaining four optodes were added for optimal coverage, resulting in eight additional channels. The source-detector distances varied between 26 and 39 mm (*M* = 34 mm).

### Data analysis

Statistical analyses were conducted using SPSS 21 (IBM Corp., Armonk, NY, USA) if not specified otherwise. The data was analysed with respect to the four n-back levels (0-, 1-, 2-, 3-back) and the two presentations of each n-back level in order to rule out confounding time-on-task effects. Bonferroni-corrected paired t-tests for each measure showed no significant differences between the two presentations of each n-back level, except for F-ISA, indicating successful randomization and no significant time-on-task effect. Therefore, the data was averaged for each n-back level except for F-ISA, where an additional analysis over time was conducted. One participant wrongly performed a 2-back instead of 3-back during both presentations of this n-back level. Their resulting missing values for the 3-back condition were replaced by the mean of the variable for ISA, performance and EEG data. For all analyses of variance (ANOVAs), sphericity was checked and, in case of violation, Greenhouse–Geisser corrected values are reported.

#### Subjective data

Concerning task difficulty, ISA values were analysed using one-way (4 n-back levels) repeated measures ANOVAs. F-ISA values were analysed with a 4 (n-back levels) × 2 (first, second presentation) repeated measures ANOVA. For further analysis of a possible time-on-task effect, F-ISA values were analysed using a one-way (8 blocks) repeated measures ANOVA. In addition, KSS values before and after the experiment were compared using a paired t-test.

#### Performance data

For the monitoring task, reaction times were analysed using a one-way (4 n-back levels) repeated measures ANOVA. For the n-back task, performance (in percent) was analysed using a one-way (4 n-back levels) repeated measures ANOVA. Performance was computed for each block as the ratio of correct responses to all responses. If participants showed more than one response per heading command (i.e. changed the heading multiple times), this was counted as uncertainty and therefore the response to this heading command as incorrect.

#### Physiological data

EEG data was pre-processed in BrainVision Analyzer 2.2 (Brain Products GmbH, Gilching, Germany). The data was down-sampled to 256 Hz, re-referenced to average and bandpass-filtered between 0.5 and 40 Hz using a 4th order IIR filter and an additional notch filter at 50 Hz to remove remaining line noise. Artefacts were removed by semi-automatic inspection and ocular correction performed via ICA. Data was divided in blocks beginning with the first reaction per block, e.g. first stimulus for a 0-back condition, second for 1-back etc., because the full working memory load was not yet reached during the first stimuli that required no reaction. Each block was segmented in epochs of 2 s with 25% overlap. Power Spectral Density was computed using Fast Fourier Transformation with a Hanning window with 10% overlap and averaged for each block. Data was exported as raw sum (µV^2^/Hz) for the theta (4–8 Hz), alpha (8–13 Hz) and beta (13–30 Hz) band for electrodes Fz, F3, F4, Pz, P3, P4, and ln-transformed to account for skewness. The frontal electrodes were analysed for the theta band, and the parietal electrodes for the alpha and beta band. For each frequency band, a one-way (n-back level) repeated measures MANOVA, using Pillai’s Trace *V*^62^ with subsequent univariate ANOVAs for each electrode of interest was computed.

fNIRS data was pre-processed and analysed in NIRS Brain AnalyzIR toolbox for MATLAB^63^. Raw voltage data was down-sampled to 4 Hz, converted to optical density and the relative concentration of oxygenated and deoxygenated haemoglobin was calculated using the modified Beer–Lambert Law^64^. The data was divided in blocks beginning with the first reaction per block and entered into a two-level general linear model (GLM) using the gamma hemodynamic response function^65^. The four n-back levels were entered as predictors. On the subject level, the short-distance channels were included as additional predictors in order to statistically control physiological confound and motion artefacts^66^. On the group level, a pre-whitening algorithm (AR-IRLS^67^) was used to correct for serial autocorrelation. The values of the GLM coefficient beta for the n-back levels per subject were entered into a mixed-effects model with a fixed intercept for each experimental condition and a random intercept for the subjects. The obtained beta values for the conditions were contrasted using t-tests, and *p* values corrected using the false-discovery rate (FDR^52^) to account for multiple comparisons. Two regions of interest (ROIs) were defined and tested post-hoc.

## Supplementary Information

Supplementary Information.

## Data Availability

The data and scripts for analysis are publicly available at PsychArchives: Data: http://dx.doi.org/10.23668/psycharchives.5291, Scripts: http://dx.doi.org/10.23668/psycharchives.5292.
