# Supplementary material for: Investigating mental workload-induced changes in cortical oxygenation and frontal theta activity during simulated flights
Source: Sci Rep. 2022 Apr 19;12:6449. doi: 10.1038/s41598-022-10044-y (PMC9018717; doi:10.1038/s41598-022-10044-y)
Supplement: Supplementary file 1 — Supplementary Information. [file 41598_2022_10044_MOESM1_ESM.pdf]

Supplements

Comparison		Lateralisation	Channel	β (SE)	*t*	*p*-value
0 vs.	1	left	AF7 - AFF5h	4.05 (1.40)	2.90	.026
			AFF3h - FFC3h	- 8.76 (2.49)	- 3.52	.006
		right	FFC4h - FFC2h	- 5.59 (1.82)	- 3.08	.016
	2	left	AF7 - AFF5h	4.92 (1.44)	3.43	.008
			FFC5h - AFF5h	6.52 (2.40)	2.71	.038
	3	left	AF7 - AFF5h	9.33 (1.45)	6.44	< .001
			FFC5h - AFF5h	15.62 (2.44)	6.39	< .001
		right	AFF6h - AF8	5.46 (1.63)	3.34	.009
			AFF6h - AFF4h	9.54 (2.28)	4.18	.001
1 vs.	3	left	AF7 - AFF5h	5.28 (1.44)	3.67	.004
			FFC5h - AFF5h	10.29 (2.51)	4.11	.001
		right	AFF6h - AF8	5.18 (1.68)	3.09	.016
			FFC4h - FFC2h	5.15 (1.90)	2.71	.038
2 vs.	3	left	AF7 - AFF5h	4.41 (1.46)	3.02	.019
			FFC5h - AFF5h	9.10 (2.50)	3.64	.004
		right	FFC4h - FFC6h	7.46 (1.93)	3.86	.002

Table S1. Significant haemodynamic results for channel-wise t-contrasts in Hbo. N = 35, df = 135, *p*-values FDR-corrected.

Comparison		Lateralisation	Channel	β (SE)	*t*	*p*-value
0 vs.	1	right	AFF6h - AF8	- 1.91 (0.66)	- 2.88	.026
			FFC4h - FFC6h	- 5.13 (0.90)	- 5.73	< .001
	2	left	AF7 - AFF5h	- 2.57 (0.61)	- 4.18	.001
			AFF3h - AFF5h	- 4.68 (0.86)	- 5.46	< .001
			FFC5h - AFF5h	- 3.28 (1.03)	- 3.19	.013
			FFC5h - FFC3h	- 3.18 (0.98)	- 3.24	.012
		right	AFF6h - AF8	- 2.11 (0.67)	- 3.14	.014
			AFF6h - AFF4h	- 3.51 (1.12)	- 3.14	.014
			AFF6h - FFC6h	- 4.00 (1.25)	- 3.19	.013
			FFC4h - FFC6h	- 6.89 (0.92)	- 7.51	< .001
	3	left	AF7 - AFF5h	- 3.31 (0.62)	- 5.33	< .001
			AFF3h - AFF5h	- 2.48 (0.86)	- 2.87	.026
			FFC5h - FFC3h	- 3.41 (1.01)	- 3.36	.009
		right	AFp2 - AFF4h	- 2.24 (0.86)	- 2.59	.049
			AFF6h - AF8	- 4.95 (0.70)	- 7.10	< .001
			AFF6h - AFF4h	- 3.16 (1.14)	- 2.78	.033
			AFF6h - FFC6h	- 4.37 (1.26)	- 3.46	.007
			FFC4h - FFC6h	- 6.73 (0.96)	- 7.04	< .001
1 vs.	2	left	AFF3h - AFF5h	- 3.32 (0.84)	- 3.94	.002
		right	AFF6h - AFF4h	- 2.96 (1.11)	- 2.65	.043
	3	left	AF7 - AFF5h	- 2.25 (0.61)	- 3.69	.004
		right	AFF6h - AF8	- 3.04 (0.70)	- 4.34	.001
			AFF6h - FFC6h	- 3.35 (1.27)	- 2.64	.043
2 vs.	3	right	AFF6h - AF8	- 2.84 (0.70)	- 4.04	.001

Table S2. Significant haemodynamic results for channel-wise t-contrasts in Hbr. N = 35, df = 135, *p*-values FDR-corrected.

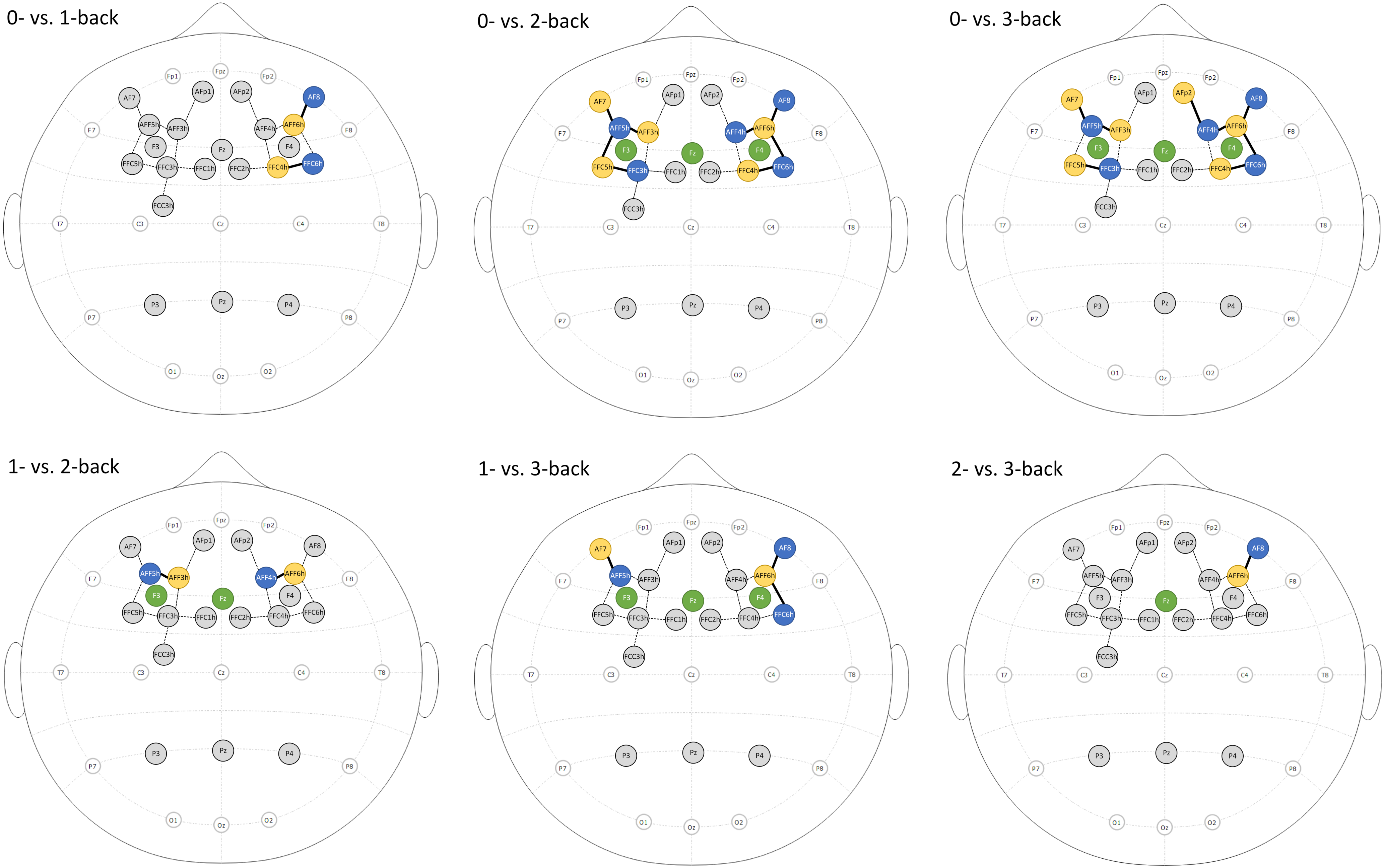

Figure S1. Overview of significant channels ($p < .05$) for each n-back level comparison. Grey circle = channel used in analysis; green circle = significant EEG electrode; yellow and blue circles connected by bold line: significant fNIRS channel.
